# Perceptions of first and third year medical students on self-study and reporting processes of problem-based learning

**DOI:** 10.1186/1472-6920-4-16

**Published:** 2004-09-22

**Authors:** Berna Musal, Yucel Gursel, H Cahit Taskiran, Sema Ozan, Arif Tuna

**Affiliations:** 1 Medical Education Department, Dokuz Eylul School of Medicine, Inciralti, Izmir, Turkey

## Abstract

**Background:**

The objective of this study is to investigate the perceptions of first and third year medical students on self-study and reporting processes of Problem-based Learning (PBL) sessions and their usage of learning resources.

**Methods:**

The questionnaire applied to the students consisted of; questions about students' perceptions on searching and preparing phases of the self-study process, the breadth and depth of discussion during reporting phase and the usage of learning resources.

**Results:**

First-year students spent more time for self-study and more highly rated the depth of discussion compared to third-year students. The searching and preparing phases of the self-study process were considered as statistically important factors strongly influencing the breadth and depth of discussion during the reporting phase. The effect of extensiveness of searching on the depth of discussion was negative among the first-year students, and positive among third-year students.

**Conclusions:**

The relative shortness of third-year students' self-study periods can be related to their mental weariness, decreased motivation or first-year students' slowness in accessing appropriate resources. The third-year students' more frequent use of textbooks may be due to the improvement of their abilities in reaching relevant learning resources. The findings implied that the increase in students' PBL experience paralleled the development of their discussion skills using different learning resources.

## Background

The institution of the present study, Dokuz Eylul University School of Medicine (DEUSM), has been implementing Problem-based Learning (PBL) in its undergraduate curriculum since the 1997–1998 academic year. The duration of undergraduate medical education is six years. PBL is the principal educational strategy in the first three years. Task-based learning strategy was adopted as an educational strategy for clerkships in the 2000–2001 academic year. During the first three years of undergraduate education, PBL sessions are the main focus of a modular structure.

The three objectives of PBL are; acquisition of essential knowledge, use of knowledge in clinical contexts and self-directed learning [1]. In a PBL programme, the students use a seven-step procedure to structure their activities. This procedure consists of clarifying vague phrases and concepts in the problem, defining the problem, analysing the problem on the basis of prior knowledge, arranging the proposed explanations, formulating learning objectives, trying to fill in the knowledge gaps by means of self study and reporting the findings in the group [2]. Individual study is an essential step of information processing approach to learning. The group members individually collect information with respect to the objectives [3].

PBL curriculum emphasises the development of self-regulating skills. Rather than being passive recipients of information, students are expected to be actively involved [4]. The diversification of learning resources and their access routes had a considerable impact on professional skills development. Due to the fact that medicine requires lifelong learning, it has become important for students to discover learning resources on their own and to interpret their findings. The use of a variety of resources necessitates the processing of information through critical self-directed inquiry. Important components of a PBL curriculum are self-directed learning and students' investigation of learning objectives. They support deep approaches to learning. Compared to traditional lecture-based curriculum, students in a PBL curriculum use a greater number and variety of resources [5].

In PBL, students are encouraged to take substantial responsibility for their learning. Small group discussions stimulate independent and active learning. They guide the students during their independent and self-directed learning. During a PBL group session, limited only by the boundaries of their prior knowledge, students try to clarify the issues being discussed. The issues that cannot be explained thoroughly are formulated as student-generated learning objectives which will guide the students during their independent and self-directed learning.

During the searching phase of individual study, students are expected to refer to different learning resources and search for literature relevant to their learning issues. The findings are evaluated and prepared for the group discussion. The extensiveness of different learning resources used is an indicator of students' self-directed learning skill. The consultation of diverse information sources influences the breadth and depth of discussion of the tutorial group during the reporting phase [6].

The iteration of self-directed learning periods leads the students to organise and review the learning resources critically. Through this critical approach, a major educational objective of PBL, the learning resources can be efficiently and effectively accessed and relevant information can be elaborated to form the theoretical basis leading to the solution of the problem [1]. Students learn most effectively when using a variety of information resources. Therefore, the provision of adequate resources meeting the needs of different learning styles is important. Students' accessibility to these learning resources may be limited if they are preserved at different locations under the management of different departments [7]. Since the beginning of the curriculum change process in Dokuz Eylul University School of Medicine (DEUSM), a special emphasis has been given to the diversification and improvement of learning resources, and facilities have been improved to meet the learning needs of PBL students. During the first three academic years of DEUSM, approximately 30–35% of the weekly schedule is allocated to students' self-study periods. Several types of learning resources such as the library, the Learning Resources Centre and a computer laboratory with Internet access are available to students. In addition, the staff teachers provide appointment-based scientific counselling upon request. The library, offering a wide variety of printed material like textbooks and periodicals is open on office days and weekends between 8:30 a.m.–11:00 p.m. The Learning Resources Centre, an interactive learning environment inaugurated in the 2001–2002 academic year, is open on office days between 8:30 a.m.–7:00 p.m. The learning resources of the centre are; CD-ROMs, video-tapes, microscopes, histology and pathology slides, models & mannequins, posters and computers with Internet access. The centre's exhibitions of learning material are synchronous with the PBL modules being implemented.

During the first week of their medical education, first-year students attend a PBL orientation course. This course consists of basic principles of PBL, student and tutor roles and presentation of available learning resources in DEUSM. All tutors are initially required to take a PBL course and regularly attend weekly tutor meetings [8]. The tutor profile of the first three years is similar. Faculty members from all existing preclinical and clinical departments fulfil tutor role in PBL groups for determined periods of time during an academic year. Some alterations observed by tutors in preparing and reporting processes between novice and experienced PBL students led us to plan the present study.

The research questions of this study are;

√ What were the differences between first and third-year students' perceptions with respect to self-study and reporting processes of PBL?

√ What were the length of students' self-study times and their usage of learning resources?

√ What was the overall and year-specific impact of self-study process on the breadth and depth of discussion during the reporting phase?

The objective of the present study is to investigate and compare the perceptions of first and third-year PBL students on self-study and reporting processes and their usage of learning resources.

## Methods

The first-year students of DEUSM who were recently introduced to PBL programme and completed their first semester and the third-year students who had a two and a half year experience in the PBL programme were included in this study.

The questionnaire consisted of questions about students' perceptions on searching and preparing phases of the self-study process, the breadth and depth of discussion during reporting phase and the usage of learning resources. The questionnaire implemented and tested for validity and reliability in Maastricht University [6] was translated into Turkish, using expressions appropriate for our study group. Two questions, one on self-study time and the other on the usage of learning resources were added (Appendix 1).

The participants reflected their perceptions of 22 questionnaire items, grouped under the headings of searching and preparing phases of self-study process, breadth and depth of discussion during the reporting phase, on a five-point scale ranging from 1 = totally disagree to 5 = totally agree. The points attributed to the items of the scale were evaluated between 1 to 5 points (1 = minimum, 5 = maximum).

The pilot study of the questionnaire was applied to 10 medical students and favourable results were obtained. At the beginning of a PBL session in February 2002, the questionnaire was distributed to first and third-year students and collected 15 minutes later. Before the application of the questionnaire, the purpose of the study was briefly explained to the students and their oral consents were obtained.

The response rate was 78.8% (115/146) for first-year students and 85.5% (142/166) for third-year students.

SPSS 10.0 for Windows was used and the reliability coefficient was calculated (Cronbach alpha: 0.81). Chi-Square Test was used to investigate students' frequency of use of learning resources. Independent Samples T Test was used to compare the scores of self-study and reporting processes of both classes. The effects of independent variables on the breadth and depth of discussion were analysed with Multiple Regression Analysis Test.

## Results

The weekly self-study times of the first and third-year students during a two-week module were 15.00 ± 8.83 and 11.57 ± 7.04 hours respectively (p = 0.001).

The first and third-year students' percentages of learning resources usage and statistical difference between them were respectively as follows; textbooks 24.3% and 44.4% (χ^2 ^= 11.1, p = 0.01), educational CDs 14.8% and 6.3% (χ^2 ^= 4.983, p = 0.026), lecture handouts 95.7% and 95.8% (χ^2 ^= 0.002, p = 0.962) and medical journals 8.7% and 9.9% (χ^2 ^= 0.102, p = 0.750). Lecture handouts were used very frequently. It was found that third-year students referred more frequently to textbooks than first-year students.

The scores reflecting students' perceptions of statements regarding learning issue driven searching and extensiveness of searching ranged between 3.4–3.7 out of five (Table 1). Third-year students' scores for the same statements were higher than those of first-year students' (p = 0.034, p = 0.009). First-year students' average scores on explanation-oriented preparing of learning issues were higher than those of third-year students' (p = 0.015).

**Table 1:** First and third-year students' average scores regarding self-study process

**Titles**	First-year students average score* ± SD	Third-year students average score* ± SD	Statistical analysis**
Learning issue driven preparing	3.5 ± 0.8	3.7 ± 0.8	P = 0.034
Extensiveness of searching	3.4 ± 0.9	3.7 ± 0.9	P = 0.009
Explanation-oriented preparing	3.5 ± 0.6	3.3 ± 0.7	P = 0.015

* (1 = minimum, 5 = maximum)

** Independent samples T test.

The scores reflecting students' perceptions on statements regarding the discussion of learning objectives during the reporting phase of a PBL session, ranged between 3.2–3.7 out of five. First-year students' average scores on the depth of discussion were higher than those of third-year students' (p = 0. 000) (Table 2).

**Table 2:** First and third-year students' average scores on discussion during reporting phase

**Titles**	First-year students' average score* ± SD	Third-year students' average score* ± SD	Statistical analysis**
Breadth of discussion	3.3** ± **0.8	3.2** ± **0.9	P = 0.345
Depth of discussion	3.7** ± **0.7	3.4** ± **0.8	P = 0.000

* (1 = minimum, 5 = maximum)

** Independent samples T test.

The effects of learning issue driven searching, extensiveness of searching and explanation oriented preparing on the breadth and depth of discussion were analysed with regression analysis (Table 3).

**Table 3:** The effects of the independent variables on the breadth and depth of discussion (Multiple Regression Analysis)

	**R^2^**	**β**	**t**	**F**
	
***Dependent variable: Breadth of discussion***				
**Searching process**				
Learning issue driven searching		0.21	2.97*	
Extensiveness of searching		0.07	0.96	
**Preparing process**	**0.09**			6.976*
Explanation-oriented preparing		0.14	1.89*	
***Dependent variable: Depth of discussion***				
**Searching process**				
Learning issue driven searching		0.26	3.87*	
Extensiveness of searching		-0.07	-1.08	
**Preparing process**	**0.15**			12.377*
Explanation-oriented preparing		0.26	3.87*	

*p < 0.05

The 9% change in the breadth of discussion was explained with searching and preparing phases. Learning issue driven searching with the highest β value was the most important and statistically significant factor influencing the breadth of discussion. The impact of explanation oriented preparing on the breadth of discussion was also statistically significant (Table 3).

The 15% change in the depth of discussion was explained with searching and preparing phases. It was found that learning issue driven searching and explanation oriented preparing were the most important and statistically significant factors influencing the depth of discussion. The extensiveness of searching had a statistically insignificant negative effect on the depth of discussion (Table 3).

Regression analysis was separately carried out for both classes. Excluding the extensiveness of searching, other findings were similar. Extensiveness of searching had a statistically negative effect on the depth of discussion among first-year students (β = -0.28, t = -2.680, p = 0.009), but a positive effect among third-year students (β = 136, t = 2.198, p = 0.030).

## Discussion

A probable explanation for the relative shortness of third-year students' self-study times compared with those of first-year students' was third-year students' mental weariness due to continuous and intensive effort in reaching learning objectives. Another probable explanation was first-year students' slowness in accessing appropriate resources due to their lack of familiarity with self-directed learning.

It was found that first and third-year students frequently used lecture handouts. The reasons for the high frequency of use of handouts were considered as their wide availability due to their provision at the end of lectures that are limited to one hour a day. In order not to hinder the curiosity of students and their motivation for self-directed learning, handouts are designed as brief outlines prepared by lecturers and include topic titles, schemata, algorithms and tables.

Since the Learning Resources Centre was inaugurated in 2001, third year students could only use it during their second and third years, whereas first-year students started using it since the beginning of their medical education. More frequent use of Learning Resources Centre CDs by first-year students may be related to their early encounter with these learning facilities.

Third-year students' more frequent use of textbooks may reflect the development of their ability in reaching essential resources.

Third-year students' higher ratings for learning issue driven searching and extensiveness of searching are consistent with the general expectation stating that experienced students can search more learning resources [4]

The first-year students attributed higher scores to statements regarding explanation oriented preparing (Table 1). This finding may be explained with third-year students' mental weariness due to continuous and intensive efforts since the beginning of their medical education or first-year students' eagerness to adapt to a new educational system.

In the Maastricht study, first-year students' average scores for learning issue driven searching, extensiveness of searching and explanation oriented searching were 3.1 ± 0.3, 2.8 ± 0.4 and 3.2 ± 0.3 respectively [6].

The scores of the entire study group attributed to breadth and depth of discussion during the reporting phase varied between 3.2 ± 0.9 and 3.7 ± 0.7. Although third-year students were expected to become more competent in PBL discussions, third-year students' scores, especially the ones attributed to the depth of discussion, were lower than those of first-year students' (Table 2). This finding is consistent with Cohen's view that the students who gain experience in cooperative study are inclined to limit their efforts to share and explain the information they gather [9]. Higher scores of first-year students on the depth of discussion may also be interpreted as a reflection of their adaptation to the PBL system. In the Maastricht study, average scores attributed by first-year students to the breadth and depth of discussion were 3.0 ± 0.4 and 3.4 ± 0.5 respectively [6]

The results of this study showed that the breadth of discussion during reporting phase was affected by the searching and preparing phases of the self-study process. Similarly, it was understood that the depth of discussion during the reporting phase was affected by the searching and preparing phases of self-study process. Learning issue driven searching "*using learning objectives as study references*" and explanation oriented preparing "*studying and summarising learning resources at an appropriate level to be shared with other students during PBL session*", directly influenced the breadth and depth of discussion.

It was observed that students' searching of different resources (extensiveness of searching), though not statistically significant, negatively affected the depth of discussion. During a PBL session, in the presence of different resources, in-depth understanding of newly acquired information requires a well-structured discussion. This may be difficult for first-year students due to their lack of familiarity with PBL and self-directed learning. In the group session, first-year students may encounter difficulties while tutoring a discussion based on several resources [6]. When regression analysis was separately carried out for both classes, it was observed that extensiveness of searching had a statistically negative effect on the depth of discussion among first-year students, but a positive effect among third-year students. These findings imply that the increase in students' PBL experience paralleled the development of their discussion skills using different learning resources.

The design of the present study based on students' perceptions was its main limitation. The assumptions regarding the causes of differences between first and third-year students' perceptions were based on the PBL experience of authors.

## Conclusions

First-year students' longer self-study times, higher explanation oriented preparing scores, and higher depth of discussion scores compared with the scores of third-year students were considered as research questions which need to be investigated in future studies.

## Competing interests

None declared.

## Authors' contributions

**BM **carried out research design, statistical data analysis, data evaluation, research report, **YG **carried out research design, data evaluation, research report, **HCT **carried out data evaluation and research report, **SO **carried out data evaluation and contributed to the research report, **AT **contributed to research design, data collection and data evaluation. All authors read and approved the final manuscript.

## List of abbreviations

DEUSM: Dokuz Eylul School of Medicine

PBL: Problem-based Learning

## Appendix

Self-study and Reporting Processes Questionnaire

**Year: **1 □ 3 □

• **What is your weekly average study time in a two-week module?**

(..............) hours.

• **What kind of resources do you use during your self-study process?**

() Textbook () Lecture handouts () Others .....................

() Educational CDs () Medical journals

• **Please indicate your level of agreement with the following statements about self-study process, by rating them between 1-to-5.**

(1 = totally disagree, 2 = disagree, 3 = neutral, 4 = agree, 5 = totally agree)

1 2 3 4 5

*Search Phase *

Learning Issue Driven Searching

1. I use the learning issues as a starting point for my search and search the resources accordingly.

During studying

2. I always check the learning issues to decide whether I study deep enough.

3. I remain attached to the learning issues.

4. I check the learning issues to decide whether the resources I study fully cover the learning issues.

5. I use the learning issues as a guide while studying the resources step by step.

Extensiveness of searching

6. When searching the resources, I try to evaluate the relevancy of different books with the subject to be studied.

7. When searching the resources, I try to compare different resources about the same subject.

8. I spent a lot of time and effort on searching the resources before I start studying

*Preparing phase *

Explanation Oriented

9. I study the subjects such that I can explain them without looking at the resources.

10. I study the subjects such that I can comment about the theories being discussed

11. I study such that I can explain the content of the resource in my own words.

12. I study such that I know what needs to be discussed in each learning issue.

13. I study by making summaries of the selected resources.

14. I study by making notes and algorithms.

*Reporting Phase *

Breadth of Discussion

15. Many different issues/findings are discussed.

16. When a student from the group reaches an information not included in the learning issues explains it to others.

17. The members of the group question different aspects of the resources.

18. Different/contradicting resources are compared.

Depth of Discussion

19. During discussions new concepts are discussed and explained in detail.

20. The issues are discussed in depth.

21. The problems of the scenario are questioned and clarified using the newly learned knowledge.

22. The discussion in the reporting makes very useful contributions to newly learned knowledge in the self-study process.

## Pre-publication history

The pre-publication history for this paper can be accessed here:


